# Ameliorative impact of sacubitril/valsartan on paraquat-induced acute lung injury: role of Nrf2 and TLR4/NF-κB signaling pathway

**DOI:** 10.1007/s00210-025-03785-w

**Published:** 2025-01-27

**Authors:** Nourhane M. Elemam, Manar A. Nader, Marwa E. Abdelmageed

**Affiliations:** 1 Department of Pharmacology and Toxicology, Faculty of Pharmacy, Mansoura University, Mansoura, 35516 Egypt; 2 Department of Pharmacology and Toxicology, Faculty of Pharmacy, Mansoura National University, Gamasa, 7731168 Egypt

**Keywords:** PQ, ARNI, Nrf2, TLR4, NF-κB

## Abstract

Herbicides such as paraquat (PQ) are frequently utilized particularly in developing nations. The present research concentrated on the pulmonary lesions triggered by PQ and the beneficial effect of the angiotensin receptor neprilysin inhibitor (ARNI), sacubitril/valsartan, against such pulmonary damage. Five groups of rats were established: control, ARNI, PQ (10 mg/kg), ARNI 68 + PQ, and ARNI 34 + PQ. Following euthanasia, lungs were isolated and subjected to a histopathological test, and the ELISA technique was used to evaluate oxidative stress biomarkers, toll-like receptor 4 (TLR4), nuclear factor erythroid 2–related factor 2 (Nrf2), phosphatidylinositol-3-kinase (PI3K), phosphorylated protein kinase B (p-AKT), and inflammatory markers: nuclear factor kappa B p65 subunit (NF-κB p65), tumor necrosis factor α (TNFα), and interleukin 1beta (IL-1β). In conjunction with abnormally high levels of malondialdehyde (MDA) and inducible nitric oxide synthase (iNOS), the PQ group also displayed low levels of reduced glutathione (GSH) and total antioxidant capacity (TAC). Additionally, TLR4, PI3K, and p-AKT were significantly elevated together with unusually low level of Nrf2. Moreover, inflammatory biomarkers, NF-κB p65, TNFα, and IL-1β, were abnormally elevated. Meanwhile, ARNI-treated groups reversed all alterations precipitated by PQ in a dose-dependent manner. ARNI could mitigate pulmonary damage triggered by PQ *via* potential antioxidant anti-inflammatory qualities.

**Graphical Abstract:**

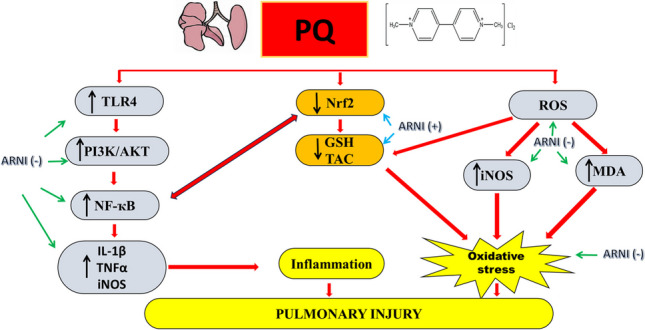

## Introduction

In today’s world, poisoning with agricultural toxins constitutes a major concern especially in developing countries (Anaeigoudari et al. 2015). Paraquat (PQ), also known as N, N′-dimethyl-4,4′-bipyridinium, is considered one of the most toxic herbicides to humans as exposure to such toxin may cause a variety of multiorgan failure and fatalities (Dinis-Oliveira et al. 2008; Wilks et al. 2008).

PQ was documented to be a major cause of pulmonary injury where lungs are considered the major target of PQ, where this chemical can precipitate pulmonary edema, inflammation, fibrosis, hemorrhage, and epithelial cell infiltration leading to respiratory failure which is supposed to be the main cause of death as a result of PQ intoxication (Venkatesan 2000). Additionally, PQ had the ability to accumulate in alveolar types I and II and Clara cells, leading to pulmonary concentrations that are 6 to 8-fold greater than those in the stream (Memarzia et al. 2024; Smith et al. 1983). PQ can also accumulate in the body leading to liver, heart, and multiple organ failure; at present, the treatment guidelines for patients with PQ poisoning are evolving and there is no specific treatment for PQ poisoning; most rely on supportive care that includes anti-inflammatory; and antioxidants drugs and treatment methods mainly depend on emetic induction, catharsis, gastric lavage, hemodialysis, immune regulation, and hemoperfusion (Xu et al. 2015). Despite these treatments, the strong toxicity of PQ poisoning and the lack of specific antidotes for it have resulted in mortality that is still >50%, though they do not have the ability to cure poisoning completely and new medicines are needed to prevent PQ-induced lung injury (Shahabadi et al. 2022).

Although the precise cause behind PQ toxicity is still unclear, oxidative stress is postulated to be the major cause of multiple organ damage precipitated by PQ (Memarzia et al. 2024). Peroxidative damage occurs in PQ toxicity when PQ enters the cells, where PQ^+2^ transfers electrons to O_2_ by cycles of repeated acquisition leading to the formation of O_2_^-^ along with reactive oxygen species (ROS) overproduction (Liao et al. 2017). This prementioned peroxidative cytotoxic damage leads to the activation of multiple inflammatory responses, and as mitochondria is known to be a major source of ROS, its dysfunction plays a vital role in damage induced by PQ (Liao et al. 2017).

Toll-like receptors (TLRs) are considered significant pattern recognition receptors that can recognize damage-associated molecular patterns (DAMPs) that are released due to non-infectious stimuli (Kovach and Standiford 2011; Oakes et al. 2013). Recent studies have documented that TLR4 receptors have the ability to recognize PQ-related DAMP and activate its downstream inflammatory molecules (Dong et al. 2013; Qin et al. 2016). Furthermore, the phosphatidylinositol-3-kinase (PI3K)/protein kinase B (AKT) signaling pathway has a vital role in cellular differentiation, proliferation, protein metabolism, and anti-inflammatory processes (Jiang et al. 2021; Li et al. 2024b). In recent years, studies have confirmed that the activation of such a pathway can cause pulmonary fibrosis and that the inflammatory pathogenesis of PQ toxicity may involve the activation of PI3K/AKT pathway (Gui et al. 2015; Jiang et al. 2021).

Sacubitril, a neprilysin inhibitor, and valsartan, an angiotensin receptor blocker, together make up the angiotensin receptor neprilysin inhibitor (ARNI), which was first used to treat heart failure (Roca et al. 2024). ARNI has been shown to be effective in mitigation of oxidative damage and inflammation in different studies of early diabetic nephropathy, methotrexate-related cardiotoxicity, and high-fat diet/streptozotocin-induced diabetic kidney disease (Dogan et al. 2023; Mohany et al. 2022; Pan et al. 2022).

It was found that ARNI prevented acute lung injury in cecal ligation and puncture mice by inhibiting pyroptosis of macrophages (Wang et al. 2023a). ARNI also effectively abrogated the cyclophosphamide-induced lung inflammation and fibrosis, *via* inhibition of nuclear factor kappa B (NF-κB) and mitogen-activated protein kinases (MAPK) signaling pathways (Abdel-Latif et al. 2020). Due to the documented antioxidative anti-inflammatory properties of ARNI, we hypothesize the ability of ARNI to attenuate oxidative stress and inflammation related to PQ-induced pulmonary injury in rats.

## Materials and methods

### Materials

PQ was brought from Tokyo Chemical Industry, Japan. ARNI was purchased as commercially available tablets (Entresto 50 mg) from Novartis AG (Basil, Switzerland); Ellman’s reagent, trichloroacetic acid (TCA), thiobarbituric acid (TBA), and sodium dodecyl sulfate (SDS) were obtained from Sigma Aldrich Chemical Company (St. Louis, MO, USA). All used chemicals were of highly fine analytical grade.

### Experimental animals

Male albino *Wistar* rats (180–220 g weight) were purchased from “Egyptian Organization for Biological Products and Vaccines (Vacsera CO.)” Giza, Egypt. Rats were observed for one week before the experiment started to allow for adaptation. During the experimental study, rats were kept in the animal facility at the Faculty of Pharmacy, Mansoura University, under standard environmental and nutritional conditions. The experimental design has been approved by Mansoura University animal care and use committee, under code number MU-ACUC (PHARM.MS.23.04.10) according to the guidelines outlined in the National Institutes of Health Guide for the Care and Use of Laboratory Animals (NIH publication no. 85-23, revised 2011).

### Experimental protocol

A random allocation (rats were split into five groups of six animals) was assigned to all the rats at the same time using simple (or unrestricted) randomization, which excluded all other variables:

Group I (normal control group) received carboxymethylcellulose (CMC) solution (0.5%) for 14 days and received a single injection of distilled water intraperitoneally (I.P) on day 7.

Group II (ARNI group) received 68 mg/kg of ARNI orally daily for 14 days; ARNI was dissolved in CMC solution (0.5%) (Stanko et al. 2024). To check if the chosen ARNI dosages were feasible, pilot research was carried out.

Group III (PQ group) received single dose of PQ (10 mg/kg) dissolved in distilled water I.P on day 7 (Chanyachukul et al. 2004; Ossowska et al. 2005).

Group IV (ARNI 34 + PQ group) received ARNI (34 mg/kg) orally for 14 days, and a single I.P dose of PQ (10 mg/kg) in day 7 1 h after ARNI administration (An et al. 2022).

Group V (ARNI 68 + PQ group) received ARNI (68 mg/kg) orally for 14 days and a single I.P dose of PQ (10 mg/kg) on day 7 and 1 h after ARNI administration.

On day 15 and 24 h after ARNI administration, all rats were anesthetized using sodium secobarbital (40 mg/kg, I.P); then, lungs of the rats were removed and rinsed with normal saline (0.9%). Tissues were weighed and processed as follows: The right lobe of the lung was fixed in 10% buffered formalin for histopathological analysis, while the left one was homogenized in phosphate-buffered saline (PBS, pH 7.4) and frozen at −80 °C for the assessment of oxidative stress and inflammatory biomarkers.

### Assessment of histopathological changes in pulmonary tissues

The kept pulmonary tissues in 10% formalin buffer were then embedded in paraffin wax, sectioned, stained with hematoxylin-eosin (H&E), before being viewed under light microscope (Olympus CH2, Japan) (Suvarna et al 2012), histopathological were recorded in 6 sections per slide, and three photos were taken per section. The pathologist was blinded on control, diseased, and treated groups as samples are coded by groups and relevant background material including study design and objectives are disclosed to pathologist who worked independently and utilized a semiquantitative scale to assess pathological alterations (Gibson-Corley et al. 2013). Pulmonary tissues’ histopathological scoring was set as follows: 0: no thickening of intra-alveolar septa, no bronchiolar damage, and no inflammation; 1: minimal thickening of intra-alveolar septa, rare bronchiolar damage, and infrequent perivascular inflammatory aggregates; 2: moderate thickening of intra-alveolar septa, minimal to mild bronchiolar damage, and frequent perivascular inflammation extending to alveolar septa with mild peribronchiolar inflammation; 3: severe thickening of intra-alveolar septa, moderate to severe bronchiolar damage, and dense perivascular peribronchiolar inflammation (Guo et al. 2012). The histopathological scores were recorded and analyzed, and median ± interquartile range (IQR) of six sections was calculated and presented.

### Assessment of oxidative stress biomarkers in pulmonary tissues

Total antioxidant capacity (TAC), reduced glutathione (GSH), malondialdehyde (MDA), and inducible nitric oxide synthase (iNOS) levels were estimated in pulmonary homogenates. TAC was measured using commercially accessible kit (cat number: TA 2513, Biodiagnostics Co, Egypt). GSH and MDA were estimated in pulmonary tissues using methods according to previously published literature (Abdelmageed et al. 2016a; Abdelmageed et al. 2016b). Additionally, iNOS was measured using commercially accessible kit (cat number: NBP2-80257, Novus Biologicals, Colorado, USA).

### Assessment of TLR4, PI3K, phosphorylated AKT (p-AKT), nuclear factor kappa B p65 Phospho-Ser536 subunit (NF-κB p65 (P-Ser536)), tumor necrosis factor α (TNFα), interleukin 1 beta (IL-1β), and nuclear factor erythroid 2–related factor 2 (Nrf2) in pulmonary tissues

TLR4, PI3K, and p-AKT were measured using commercially accessible ELISA kits that were performed according to basic principles previously described (Tabatabaei and Ahmed 2022): (TLR4: cat number: SEA 753Ra, Cloud-Clone-Corp, Houston USA), (PI3K: cat number: ER1910, Fine Test, Wuhan, China), and (p-AKT: cat number: MBS1600201, MyBiosource, San Diego, USA), respectively. Furthermore, NF-κB p65 (P-Ser536), TNFα, and IL-1β were measured using commercially accessible kits (NF-κB p65 (P-Ser536): cat number: MBS9511033, MyBioSource, San Diego, CA, USA), (TNFα: cat number: 438206, Biolegend, San Diego, California, USA), and (IL-1β: cat number: E0119Ra, BT LAB, Shanghai, China), respectively. Nrf2 was also measured using an ELISA kit (cat number: RD-NFE2L2-Ra, Reddot Biotech INC, Kelowna, Canada).

### Statistical analysis

Data were displayed as means ± SD. Kolmogorov-Smirnov test was used to check for data normality. GraphPad Prism V 8.0.1 (GraphPad Software Inc., San Diego, CA, USA) was used for statistical analysis and graphical depiction. For multiple comparison, parametric data were analyzed using a standard one-way ANOVA and then Tukey’s post hoc test. Non-parametric data, which were provided as the median ± IQR, were examined using the Kruskal Wallis test and then Dunn’s post hoc test. *p* < 0.05 was used to indicate statistical significance.

## Results

### ARNI attenuated PQ-induced histopathological alterations in pulmonary tissues

**Fig. 1:**
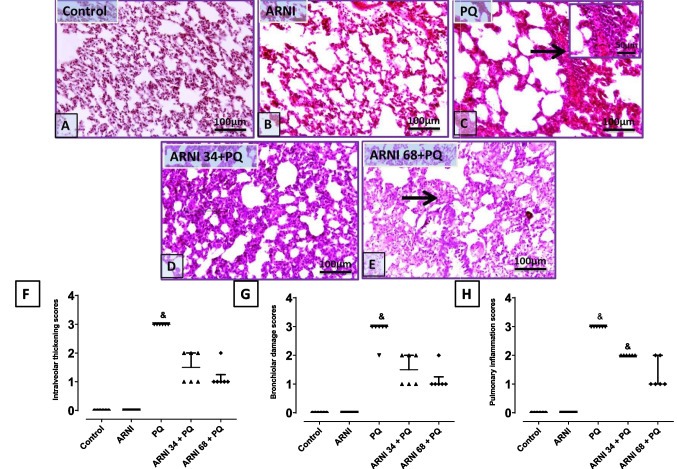
Impact of ARNI on histopathological alterations in pulmonary tissues of PQ-injected rats using H&E stain (low magnifications: X: 100 and high magnifications X: 400). **A**,** B** Pulmonary tissues of control and ARNI groups showed a normal histological appearance of lung alveoli and interalveolar septa. **C** Pulmonary tissues of PQ group showed mild expansion of interalveolar septa by numerous coalescing aggregations of mononuclear cells and numerous aggregations of lymphocytes, macrophages, fibroblast, and admixed with RBcs expanded the interalveolar septa (thin arrow). **D** Pulmonary tissues of (ARNI 34 + PQ) showed few alveolar damage with moderate expansion of interalveolar septa (thin arrow). **E** Pulmonary tissues of (ARNI 68 + PQ) showed minimal interalveolar septa thickening (thin arrow). **E**,** F**,** G** Semi-quantitative scoring of intra-alveolar thickening, bronchiolar damage, and inflammation respectively. PQ, paraquat; ARNI, angiotensin receptor neprilysin inhibitor. Data are expressed as median ± interquartile range (*n* = 6). ^&^(*p* < 0.05) vs. control group using Kruskal Wallis test followed by Dunn’s multiple comparison post hoc test

Figure 1A and B showed a normal histological appearance of lung alveoli and intra-alveolar septa in both control and ARNI groups, while Fig. 1C showed mild expansion of interalveolar septa by numerous coalescing aggregation of mononuclear cells and numerous aggregations of lymphocytes, macrophages, and fibroblasts, and admixed with RBcs expanded the interalveolar septa in PQ group. In contrast, ARNI 34 mg/kg-treated group showed few alveolar damage with moderate expansion of interalveolar septa, and ARNI 68 mg/kg-treated group showed minimal interalveolar septa thickening (Fig. 1D and E), respectively. As shown in Fig. 1F, G, and H, PQ group showed a severe increase in intra-alveolar septa thickening, bronchiolar damage, and inflammation respectively compared to control group, while ARNI (34 mg/kg and 68 mg/kg) treated group showed a marked decrease in the prementioned histopathological alterations compared to PQ group.

### ARNI attenuated oxidative stress induced by PQ in pulmonary tissues

**Fig. 2:**
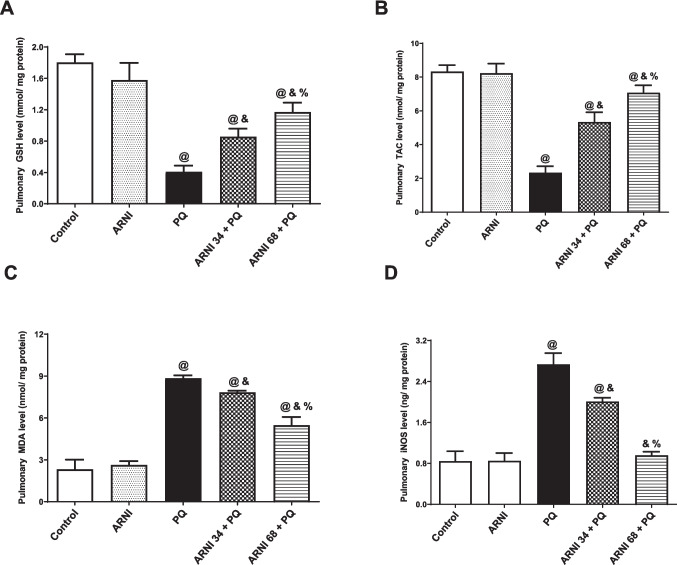
Impact of ARNI on altered oxidant/antioxidant balance in pulmonary tissues of PQ-injected rats. **A** Pulmonary reduced glutathione (GSH). **B** Pulmonary total antioxidant capacity (TAC). **C** Pulmonary malondialdehyde (MDA). **D** Pulmonary inducible nitric oxide synthase (iNOS). PQ, paraquat; ARNI, angiotensin receptor neprilysin inhibitor. The data are expressed as mean ± SD (*n* = 6). ^&, @, %^(*p* < 0.05) vs. control, PQ, and (ARNI 34 + PQ) groups, respectively, using one-way ANOVA followed by Tukey-Kramer multiple comparisons test

Figure 2A and B displayed a marked (*p* < 0.05) reduction in pulmonary GSH and TAC by 77.37% and 71.96%, respectively, in PQ group compared to control group, while (ARNI 34 + PQ) showed a significant (*p* < 0.05) elevation in GSH and TAC by 2.09 and 2.28-folds, respectively, in comparison to PQ group. Additionally, (ARNI 68 + PQ) group showed a significant (*p* < 0.05) increase in GSH and TAC by 2.87 and 3.03-folds, respectively, indicating a better antioxidant activity of the higher dose of ARNI. Both doses of ARNI showed a marked (*p* < 0.05) difference from control group, while rats treated with ARNI only showed insignificant difference from control group.

However, Fig. 2C and D displayed a marked (*p* < 0.05) increase in pulmonary MDA and iNOS by3.81 and 3.24-folds, respectively, in PQ group compared to control group. In contrast, (ARNI 34 + PQ) group showed a marked (*p* < 0.05) reduction in MDA and iNOS by 11.47% and 26.58%, respectively, in comparison to PQ group. Furthermore, (ARNI 68 + PQ) group displayed a significant (*p* < 0.05) decrease in MDA and iNOS by 37.93% and 65.04%, respectively, relative to the PQ group indicating a better antioxidant activity of the higher dose of ARNI. Both doses of ARNI displayed a significant (*p* < 0.05) difference from control group in MDA only, while rats treated with ARNI only showed insignificant difference from control group.

### Effect of ARNI on Nrf2 and TLR4 levels in pulmonary tissues

**Fig. 3:**
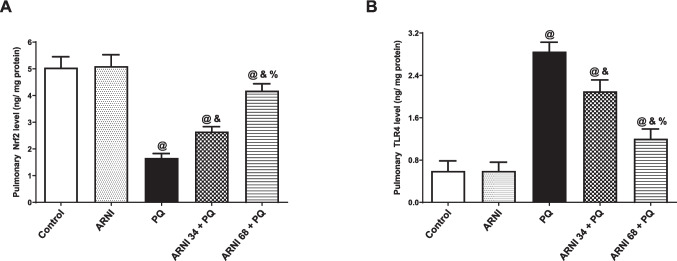
Impact of ARNI on pulmonary Nrf2 and TLR4 of PQ-injected rats. **A** Pulmonary nuclear factor erythroid 2–related factor 2 (Nrf2). **B** Pulmonary toll-like receptor 4 (TLR4). PQ, paraquat; ARNI, angiotensin receptor neprilysin inhibitor. The data are expressed as mean ± SD (*n* = 6). ^&, @, %^(*p* < 0.05) vs. control, PQ, and (ARNI 34 + PQ) groups, respectively, using one-way ANOVA followed by Tukey-Kramer multiple comparisons test

Figure 3A showed a marked (*p* < 0.05) reduction in antioxidant transcription factor, Nrf2 by 66.96% in PQ-injected rats in comparison to control group. Conversely, (ARNI 34 + PQ) and (ARNI 68 + PQ) groups showed a marked (*p* < 0.05) elevation in pulmonary Nrf2 by 1.59 and 2.51-folds, respectively, showing a better antioxidant activity of the (68 mg/kg) dose than that of (34 mg/kg). Both doses of ARNI showed a significant (*p* < 0.05) difference from control group, while rats treated with ARNI only showed insignificant difference from control group.

Contrarywise, PQ-injected rats showed a marked (*p*< 0.05) increase in pulmonary TLR4 levels by 4.81-folds compared to control group (Fig. 3B). While (ARNI 34 + PQ) and (ARNI 68 + PQ) groups showed a notable (*p* < 0.05) decrease in pulmonary TLR4 by 26.39% and 57.82%, respectively, compared to PQ group, indicating a better anti-inflammatory activity of the higher dose of ARNI. Both doses of ARNI showed a marked (*p* < 0.05) difference from control group, while rats treated with ARNI only showed insignificant difference from control group.

### ARNI attenuated PI3K and p-AKT levels in pulmonary tissues

**Fig. 4:**
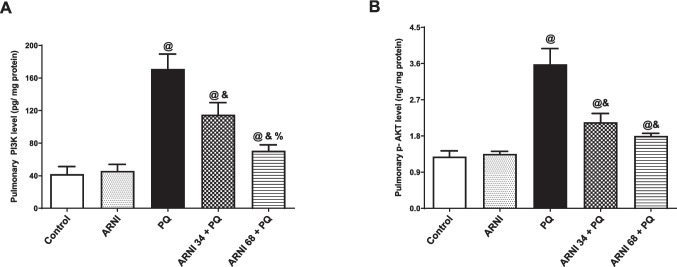
Impact of ARNI on pulmonary PI3K and p-AKT of PQ-injected rats. **A** Pulmonary phosphatidylinositol-3-kinase (PI3K). **B** Pulmonary phosphorylated protein kinase B (p-AKT). PQ, paraquat; ARNI, angiotensin receptor neprilysin inhibitor. The data are expressed as mean ± SD (*n* = 6). ^&, @, %^(*p* < 0.05) vs. control, PQ, and (ARNI 34 + PQ) groups, respectively, using one-way ANOVA followed by Tukey-Kramer multiple comparisons test

Figure 4A and B showed a significant (*p* < 0.05) elevation in pulmonary PI3K and p-AKT levels by 4.07 and 2.79-folds in PQ-injected rats compared to control group. Contrarywise, (ARNI 34 + PQ) and (ARNI 68 + PQ) groups displayed a marked (*p* < 0.05) decrease in pulmonary PI3K by 32.79% and 58.67%, respectively, compared to PQ group. Similarly, (ARNI 34 + PQ) and (ARNI 68 + PQ) groups showed a marked (*p* < 0.05) decrease in pulmonary p-AKT by 40.27% and 49.68%, respectively, compared to PQ group. These results indicated that the dose of (68 mg/kg) of ARNI had better impact in decreasing pulmonary PI3K and p-AKT levels than that of (34 mg/kg). Both doses of ARNI showed a significant (*p* < 0.05) difference from control group except in p-AKT, and rats treated with (ARNI 68 + PQ) showed insignificance from control group. Furthermore, rats treated with ARNI only showed insignificant difference from control group.

### ARNI attenuated NF-κB p65 (P-ser536), TNFα, and IL-1β levels in pulmonary tissues

**Fig. 5:**
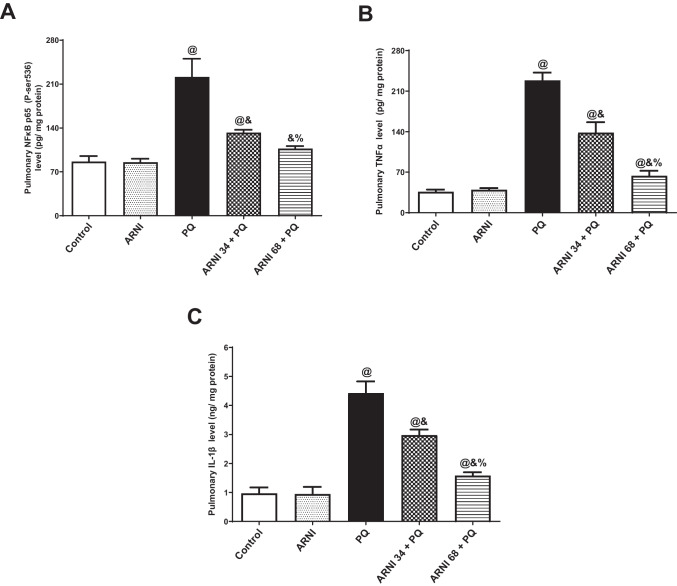
Impact of ARNI on inflammatory biomarkers in pulmonary tissues of PQ-injected rats. **A** Pulmonary nuclear factor kappa B p65 Phospho-Ser536 subunit (NF-κB p65 (P-Ser536)). **B** Pulmonary tumor necrosis factor α (TNFα). **C** Pulmonary interleukin 1beta (IL-1β). The data are expressed as mean ± SD (*n* = 6). PQ, paraquat; ARNI, angiotensin receptor neprilysin inhibitor. ^@, &, %^(*p* < 0.05) vs. control, PQ, and (ARNI 34 + PQ) groups, respectively, using one-way ANOVA followed by Tukey-Kramer multiple comparisons test

PQ injected group showed a massive (*p* < 0.05) increase in pulmonary inflammatory NF-κB p65 (P-ser536) (Fig. 5A), TNFα (Fig. 5B), and IL-1β (Fig. 5C) levels by 2.56, 6.30, and 4.58-folds, respectively, compared to control group. However, (ARNI 34 + PQ) group showed a significant (*p* < 0.05) reduction in pulmonary NF-κB p65(P-ser536), TNFα, and IL-1β by 40.17%, 39.53%, and 32.78%, respectively, compared to PQ group. Similarly, (ARNI 34 + PQ) group showed a marked (*p* < 0.05) reduction in pulmonary NF-κB p65 (P-ser536), TNFα, and IL-1β by 51.65%, 72.08%, and 64.24%, respectively, in comparison to PQ group. These results indicated that this decrease in inflammation in ARNI treated group was in a dose-dependent manner and a better anti-inflammatory activity of the higher dose of ARNI.

## Discussion

PQ toxicity is recognized to have high death rates, and according to reports, about 150,000 individuals died because of pesticide toxicity either accidentally or deliberately in 2020 (Tyrrell et al. 2021; Zhang et al. 2023). One of the most frequent cases of pesticide poisoning is PQ poisoning because of the fact that there is no specific antidote against PQ, making PQ toxicity a significant public health issue (Gawarammana and Buckley 2011; Sun and Chen 2016; Wang et al. 2017). The most prevalent organ lesion in cases of PQ toxicity is acute lung injury, which advances rapidly and may eventually lead to lung fibrosis (Wu et al. 2024). The current study showed that PQ could precipitate a massive imbalance in oxidant/antioxidant system by decreasing antioxidant, GSH and TAC, beside an abnormal increase in MDA and iNOS, also PQ-injected rats experienced a massive increase in TLR4 along with a decrease in Nrf2. Additionally, the inflammatory PI3K/AKT/ NF-κB pathway was extremely upregulated indicating an inflammatory activity which was confirmed by high levels of pulmonary of TNFα and IL-1β. Presently, the ALI induced by PQ is mainly associated with oxidative stress and inflammatory responses (Li et al. 2021). It has been shown that exposure of crude membrane preparations of rat lung to PQ resulted in a dose-dependent inhibition of neutral endopeptidase (NEP) activity, and this enzyme is considered to be critical in controlling the levels of tachykinins, and so a more sustained activity of tachykinins may be present, producing effects like cell proliferation, fluid extravasation, and bronchoconstriction (Atzori et al. 1998).

Previous investigations showed that losartan, an angiotensin (Ang) II type 1 receptor antagonist, had beneficial effects on the treatment of PQ-induced pulmonary fibrosis (Guo et al. 2015). Furthermore, it has been shown that PQ enhanced Ang II and collagen type I mRNA and protein expression and the increased chymase that converts Ang I to Ang II which is closely involved with lung fibrosis, expression in PQ-treated human lung fibroblasts, and it was confirmed *in vitro* and in an *in vivo* PQ model of lung fibrosis that chymase generates Ang II and enhances collagen expression (Lang et al. 2010).

On the other hand, the abilities of different angiotensin-converting enzyme inhibitors (ACEis) captopril (a thiol ACEi), enalapril, and lisinopril (two nonthiol ACEi) to mitigate mitochondrial toxicity induced by PQ show that captopril is a more effective antioxidant than the nonthiol ACEi due to captopril’s abilities to scavenge ROS (Ghazi-Khansari et al. 2006); furthermore, the antifibrotic effect of captopril, enalapril, and lisinopril was confirmed in various rat models of lung fibrosis (Candan and Alagözlü, 2001; Ghazi-Khansari et al. 2007; Mohammadi-Karakani et al. 2006) and hepatotoxicity (Elmi et al. 2007; Ghazi-Khansari and Mohammadi-Bardbori 2007) induced by PQ.

In context with previous studies, the current investigation provided evidence that ARNI can effectively reduce oxidative stress and inflammation-induced lung damage brought on by PQ. This protective impact was shown by the decrease in oxidative stress biomarkers and inflammatory reactions *via* the inhibition of TLR4-driven NF-κB/PI3K/AKT signaling cascade.

Regarding human studies, ARNI was shown to be more effective in reducing the risk of death from cardiovascular causes or hospitalization for heart failure than was ACE inhibition with enalapril in patients with chronic heart failure and a reduced ejection fraction; ARNI is also superior to enalapril in reducing the risk of death from any cause and reducing symptoms and physical limitations of heart failure (McMurray et al. 2014). ARNI could improve right ventricular performance and pulmonary hypertension in patients with heart failure and reduced ejection fraction (Zhang et al. 2022). On the other hand, animal studies on various lung models revealed that ARNI could effectively mitigate lung inflammation and fibrosis induced by cyclophosphamide in rats, *via* inhibition of NF-κB and MAPK signaling pathways (Abdel-Latif et al. 2020), ARNI also prevented acute lung injury in cecal ligation and puncture mice by inhibiting pyroptosis of macrophages (Wang et al. 2023a). Additionally, ARNI reduced pulmonary pressures, vascular remodeling, and right ventricular hypertrophy in a rat model of pulmonary hypertension induced by SU5416/hypoxia model (Clements et al. 2019) and ameliorated hypoxia-induced pulmonary hypertension by suppressing apoptosis, inhibiting the inflammatory response, and inhibiting the PI3K/AKT signaling pathway (Wang et al. 2023b).

Excessive free radicals are produced in the PQ redox cycle, these free radicals have the ability to extract hydrogen from polyunsaturated fatty acids leading to lipid peroxidation, which is thought to be the most significant early stage in the pathophysiological process of PQ-induced organ damage (Dinis-Oliveira et al. 2008). Lipid peroxidation can then damage mitochondrial and cell membranes leading to cell death (Choi et al. 2007). These facts are consistent with the findings of the present study, as there was a massive increase in MDA and iNOS oxidative biomarkers along with an abnormal decrease in GSH and TAC in pulmonary tissues of PQ-injected rats, while ARNI-treated groups showed a decrease in both MDA and iNOS with a considerable increase in GSH and TAC indicating a possible antioxidant effect of ARNI which was confirmed in a study of myocardial injury in rats by Refaie et al. (2024).

Nrf2 is a transcription factor that has the ability to control the transcription of the antioxidant systems as well as the regulation of detoxification enzymes (Nguyen et al. 2009). Additionally, Nrf2 can detoxify ROS, regenerate NADPH, and protect against lipid peroxidation, GSH depletion, and mitochondrial dysfunction (Liu et al. 2022). Under normal conditions, Kelch-like ECH associated protein 1 (Keap 1), which functions as an adaptor protein, is bound to Nrf2 and serves for Nrf2’s continuous ubiquitylation and degradation, while under oxidative stress conditions, Keap 1 is inactivated resulting in the translocation of Nrf2 into the nucleus where the transcription of antioxidant genes begins (Baird et al. 2014). In the present study, Nrf2 levels were markedly downregulated due to the overproduction of ROS which led to the malfunction of the antioxidant defense mechanism resulting in a disturbance in the homeostasis of this antioxidant protein, and these results are supported by the findings deduced by Zhang et al. (2023) (Zhang et al. 2023). On the other hand, ARNI group showed higher levels of Nrf2 which supports its antioxidant effect which is consistent with the findings of the previous study that demonstrated the ameliorative effects of ARNI on chronic kidney disease–induced oxidative stress and inflammation in rats (Jing et al. 2017).

TLRs are recognized as membrane-spanning receptors that are usually expressed in dendritic cells and macrophages; they are also proven to be involved in oxidative stress signal transduction network (Pahwa and Jialal 2016). Recent studies have already proved the involvement of TLRs in PQ-induced pulmonary damage, as the active form of TLR4 has the ability to stimulate NF-κB and NF-κB related inflammatory genes (Shen et al. 2017). Furthermore, TLRs have the ability to stimulate the degradation of Keap1 and subsequent translocation of Nrf2 to the nucleus and its activation (Yin and Cao 2015). On the other hand, this activation of Nrf2 has the ability to reduce TLR-mediated inflammatory responses (Mohan and Gupta 2018). The heavy generation of ROS leads to a downregulation of Nrf2 antioxidant–related transduction pathway followed by a heavy increase in TLR expression levels especially TLR4; these facts are on the same line with the findings of the present study where pulmonary TLR4 was significantly elevated in PQ-injected rats as previously mentioned in the previous studies (Liu et al. 2014; Qian et al. 2015; Shen et al. 2017). Conversely, ARNI treated groups showed lower levels of pulmonary TLR4 which is persistent with the results deduced by Khallaf et al (2023) in a previous research on cyclophosphamide-induced premature ovarian failure in rats (Khallaf et al. 2023).

PI3K/AKT pathway is a pathway that is involved in a variety of cellular processes like cell survival, growth, proliferation, and metabolism (Liu et al. 2022). The phosphorylation of PI3K leads to phosphorylation of AKT; then, p-AKT activates the expression of a series of downstream molecules including NF-κB by enhancing the transcription and translocation of NF-κB (Chu 2014; Lo et al. 2015).

Recently, it was accepted that TLR4 is closely related to PI3K/AKT pathway, as TLR4 could activate PI3K leading to phosphorylation of its downstream target, AKT (Zhao et al. 2014) In recent studies, it was proved that PI3K/AKT signaling pathway had the most gene enrichment in pulmonary tissues following PQ poisoning indicating that it is the main pathway responsible for the pulmonary inflammation seen in PQ-injected rats (Li et al. 2024a; Li et al. 2024b). On the other hand, ARNI-treated rats showed lower levels of both pulmonary PI3K and p-AKT proving its possible anti-inflammatory activity discussed before by Wang et al. (2023a, b) of the effect of ARNI in the mitigation of hypoxic pulmonary hypertension in rats (Wang et al. 2023b).

NF-κB signaling pathway has a vital role in PQ-triggered lung injury (Liu et al. 2013). NF-κB functions as a protein complex that regulates several genes related to inflammation, immunological response, and cancer and can be triggered by cytokines, free radicals, and cellular stress (Mitchell et al. 2016). In normal conditions, NF-κB dimers are associated with inhibitory proteins of IκB family and kept in dimers, but under stress conditions, IκB kinase enzymes phosphorylate IκB proteins, inducing their ubiquitination; this led to the translocation of NF-κB inside the nucleus, binding to response elements; then, the targeted genes will be transcribed then translated to be involved in the inflammatory response (Paul et al. 2018). PQ causes IκBα degradation and phosphorylation, which raises the p65 subunit of NF-κB levels in lung tissue nuclear extracts and enhances the generation of TNFα, IL-1β, and iNOS in PQ-induced lung damage (Liu et al. 2013).

Furthermore, Nrf2 plays its role in the preservation of cellular redox homeostasis by a negative regulation to NF-κB p65 *via* the reduction of ROS (Liu et al. 2022). Conversely, NF-κB p65 has the ability to prevent the activation of Nrf2 by reducing CREB binding protein (CBP), the transcriptional co-activator of Nrf2, and preventing CBP from binding to Nrf2 (Liu et al. 2008). In the current study, a significant increase in pulmonary NF-κB p65 was noticed in PQ-injected rats with a parallel increase in its downstream proinflammatory mediators, TNFα, IL-1β, and iNOS, which is on the same line with the previous study of Li et al (2021) and Liu et al (2022) (Li et al. 2021; Liu et al. 2022). On the other hand, a substantial decrease in pulmonary NF-κB p65, TNFα, IL-1β, and iNOS was marked in ARNI groups indicating for the first time the anti-inflammatory effects of ARNI that could decrease pulmonary inflammation precipitated by PQ.

Significant limitations of this study include the absence of data about bronchoalveolar lavage fluid cell count. We have not performed invasive evaluation of molecular pathways involved in oxidative stress. Although our data showed that ARNI treatments had a potential effect on lung injury induced by PQ in rats, the impact of ARNI treatment on various organ damage induced by PQ awaits further exploration.

## Conclusion

**Fig. 6:**
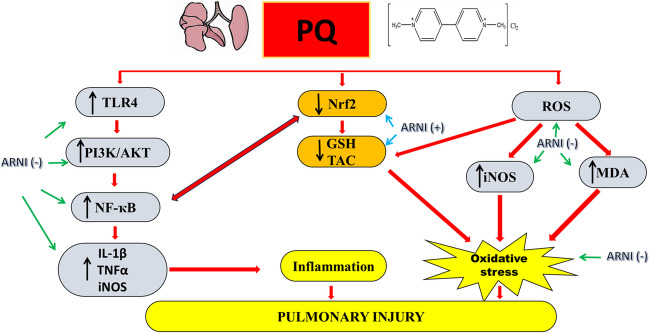
Schematic diagram of potential mechanisms by which ARNI may attenuate pulmonary injury precipitated by PQ. PQ, paraquat; ARNI, angiotensin receptor neprilysin inhibitor; ROS, reactive oxygen species; iNOS, inducible nitric oxide synthase; MDA, malondialdehyde; GSH, reduced glutathione; TAC, total antioxidant capacity; IL-1β, interleukin 1beta; TNFα, tumor necrosis factor α; NF-κB, nuclear factor kappa B; TLR4, toll-like receptor 4; PI3K, phosphatidylinositol-3-kinase; p-AKT, phosphorylated protein kinase B; Nrf2, nuclear factor erythroid 2–related factor 2

In conclusion, the *in vivo* experiment conducted in the current investigation demonstrated that ARNI could downregulate oxidative stress and inflammation induced by PQ in rats’ lungs *via* the PI3K/AKT/NF-κB pathway mediated TLR4 (Fig. 6). However, further research is needed to confirm the pulmonary protective effect of ARNI clinically and to explore additional molecular pathways to provide additional insights into the functional roles of ARNI during the treatment of ALI.

## Data Availability

All source data for this work (or generated in this study) are available upon reasonable request.
